# The Longitudinal Association Between Cardiovascular Risk and Cognitive Function in Middle-Aged and Older Adults in China: A Nationally Representative Cohort Study

**DOI:** 10.3389/fcvm.2020.560947

**Published:** 2020-10-19

**Authors:** Wei Hua, Jianhua Hou, Taiyi Jiang, Bin Su, Jiangning Fu, Runsong Sun, Biru Chang, Wei Xia, Hao Wu, Tong Zhang, Caiping Guo, Wen Wang

**Affiliations:** ^1^Center for Infectious Diseases, Beijing Youan Hospital, Capital Medical University, Beijing, China; ^2^Beijing Key Laboratory for HIV/AIDS Research, Beijing, China; ^3^Institute of Psychology, Chinese Academy of Sciences, Beijing, China; ^4^State Key Laboratory of Cognitive Neuroscience and Learning, Beijing Normal University, Beijing, China; ^5^Department of Psychology, Research Institute for International and Comparative Education, Shanghai Normal University, Shanghai, China

**Keywords:** cardiovascular risk, cognition, longitudinal study, older adult, national representative data set

## Abstract

**Objective:** China has the largest population living with dementia, causing a tremendous burden on the aging society. Cardiovascular disease (CVD) may trigger a cascade of pathologies associated with cognitive aging. We aim to investigate the association between cardiovascular risk (CVR) factors and cognitive function in the nationally representative cohort in China.

**Methods:** Participants were recruited from 150 counties in 28 provinces via a four-stage sampling method. The outcomes included several cognitive tasks. The independent variable was a composite score of cardiovascular risk calculated from sex-specific equations. We fitted the time-lagged regression to model the association between CVR and cognition. Besides, we performed cross-group analyses to test for model invariance across sex and age. We thus constrained path coefficients to be equal across each grouping variable (e.g., sex) and compared the fit of this constrained model with an unconstrained model in which the path coefficients were allowed to vary by group.

**Results:** A total of 3,799 participants were included in the final analyses. We found that the CVR had a negative linear association with global cognition (β = −0.1, *p* < 0.01). Additionally, CVR had inverse linear associations with domain-specific measurements of memory and learning, calculation, orientation, and visual–spatial ability (all values of *p* < 0.01). Regarding sex and age moderation, males had a more pronounced association between higher CVR and worse general cognition, immediate recall, orientation, calculation, and visual–spatial ability (all values of *p* < 0.0001). In contrast, females exhibited a slightly larger negative association in delayed recall. Older participants (>65 years old) had a more pronounced association between higher CVR and worse calculation ability (*p* = 0.003).

**Conclusion:** CVD are risk factors for lower global cognition and cognitive subdomains in middle-aged and older adults in China.

## Introducton

China has the largest population of patients aged over 60 living with dementia in the world, and it has an estimated population of 9.5 million (1), causing a severe public and economic burden on the social and health care systems (2). Cardiovascular disease (CVD) is the leading cause of death in China (3). Compared to the non-Asian older adults, the prevalence of hypertension, diabetes, and hyperlipidemia in China and other Asian countries might be higher and persists upward (4–6). Worsened cardiovascular health triggers a cascade of pathologies associated with cognitive impairment and dementia in older adults (7). Most of the clinical evidence is based on non-Asian populations (8–14).

Few studies exploring the association between composite cardiovascular risk (CVR) and cognition have been conducted in Chinese or other East Asia populations. Most studies examining the specific CVR (e.g., hypertension or diabetes) and cognition decline provided the mixed evidence (15–20). Most studies lacked rigorous sampling methods (17), large sample size, longitudinal follow-ups, or detailed information for other confounders (e.g., depression symptoms) (11), leading to unreliable results.

CVRs are prone to cluster in older and middle-aged adults (11). The sex-specific cardiac and vascular aging patterns in early adulthood play an essential role in later life (21). Independent examination of specific CVR may hinder our understanding of the complex interplay of different CVRs on cognition. To date, some studies examining the association of composite CVR [e.g., Framingham Cardiovascular Risk Score (FCRS)] and cognition (e.g., episodic memory) found a significant association between composite CVR and cognitive impairment, cognitive decline, dementia, and mortality rate in Whites (8, 9, 22) and Latino (23). Despite the wide use of FCRS, some have challenged the external validity to non-Whites (10). It is an urgent need to test whether FCRS is also a useful tool among Chinese adults.

This study uses the data from a nationally representative sample in Chinese to examine the composite CVR and cognitive function. Specifically, we investigate longitudinal associations between global vascular risk and domain-specific measurements. We hypothesize that higher CVR will be associated with lower cognitive performance. We also expect that sex and age modification may present in the current sample due to the heterogeneity of age- and sex-specific trajectories in cardiac and cognitive aging.

## Methods

### Participants and Protocol

The data were drawn from the first and fourth waves of the China Health and Retirement Longitudinal Study (CHARLS), a nationally representative longitudinal survey of the older residents in China. The baseline sample was obtained via a four-stage random sampling method, covering participants from 150 counties in 28 provinces in China in 2011. The study design and sampling method's detailed information can be found in Zhao et al. (24). Only waves one (2011) and four (2015) were used in the final analyses due to the blood tests' availability in these waves. Participants were excluded if they (1) are aged younger than 50 years old, (2) did not receive fasting blood test or had no testing results on CVD factors, (3) did not respond to any cognitive tests, and (4) reported having received a diagnosis of memory-related disease (such as Alzheimer disease, Parkinson, brain atrophy, or brain cancer). This study was approved by the Institutional Review Board of Beijing Youan Hospital.

### Measurements

Cardiovascular risk was defined as a pooled score (Framingham cardiovascular risk score) using sex-specific equations combining age, systolic blood pressure and antihypertension treatment, diabetes, HDL, smoking, and total cholesterol.

Cognitive function comprised (1) a global cognitive measure, based on a pooled Z-score of immediate recall, delayed recall, figure copying, calculation, orientation, and digital-series reasoning, and (2) five domain-specific measurements. The Chinese verbal learning test is an episodic learning and memory test with two scores: (1) immediate recall, and (2) delayed recall. The summed total of correct words range from 0 to10 at each recall phase. We also adopted minus 7 and orientation tests from the Montreal Cognitive Assessment (MoCA). Considering the relatively low literacy among Chinese older adults, we adopted two overlapped pentagons instead of cubic in the figure-copying test.

Covariates in the regression model for CVR–cognition associations were education (high school or above vs. below high school), Hukou (agricultural vs. non-agricultural), marital status (married, separated, divorced, and widowed, and never married), regional distribution (western China, eastern China, and mid-China), employment status (farming, non-farming, both, unemployed) depressive symptoms (using a cutoff of 10 in CESD-10), BMI, waist-to-height ratio, and residence status (family housing vs. nursing home, hospital, or other), drinking status (>once/per month, < =once per month, no) and stroke (yes, no).

### Statistic Strategies

Our analyses were conducted in three stages. First, we generated the descriptive statistics for a sample of interest to provide detailed baseline information. We used mean and standard deviation for continuous variables and proportion for categorical variables. Second, we fitted the time-lagged regression to model the association between CVR and cognition, including autoregression models and CVR main effect models. We used three primary indices to assess the adequacy of model-data fit: the comparative fit index (CFI), the standardized root mean square residual (SRMR), and the root mean square error of approximation (RMSEA). The acceptable level of model fit was CFI higher than 0.90, RMSEA lower than 0.08, and SRMR lower than 0.08 (25). Third, we performed cross-group analyses to test for model invariance across sex and age. Specifically, we constrained path coefficients to be equal across each grouping variable (e.g., sex) and compared the fit of this constrained model with an unconstrained model in which the path coefficients were allowed to vary by group. Model invariance can be assumed when the constrained model does not yield substantially poorer fit to the data than does the unconstrained version. Two indexes were used to compare the fit of the constrained and unconstrained models, the scaled difference in Satorra–Bentler (S–B) χ^2^ statistic and the difference in CFI values between models (26). The two models can be judged as invariant across moderator variables where their differences in fit do not reach the criterion for practical significance (ΔCFI differences > 0.01) (26).

## Results

### Study Characteristics

**Figure 1:**
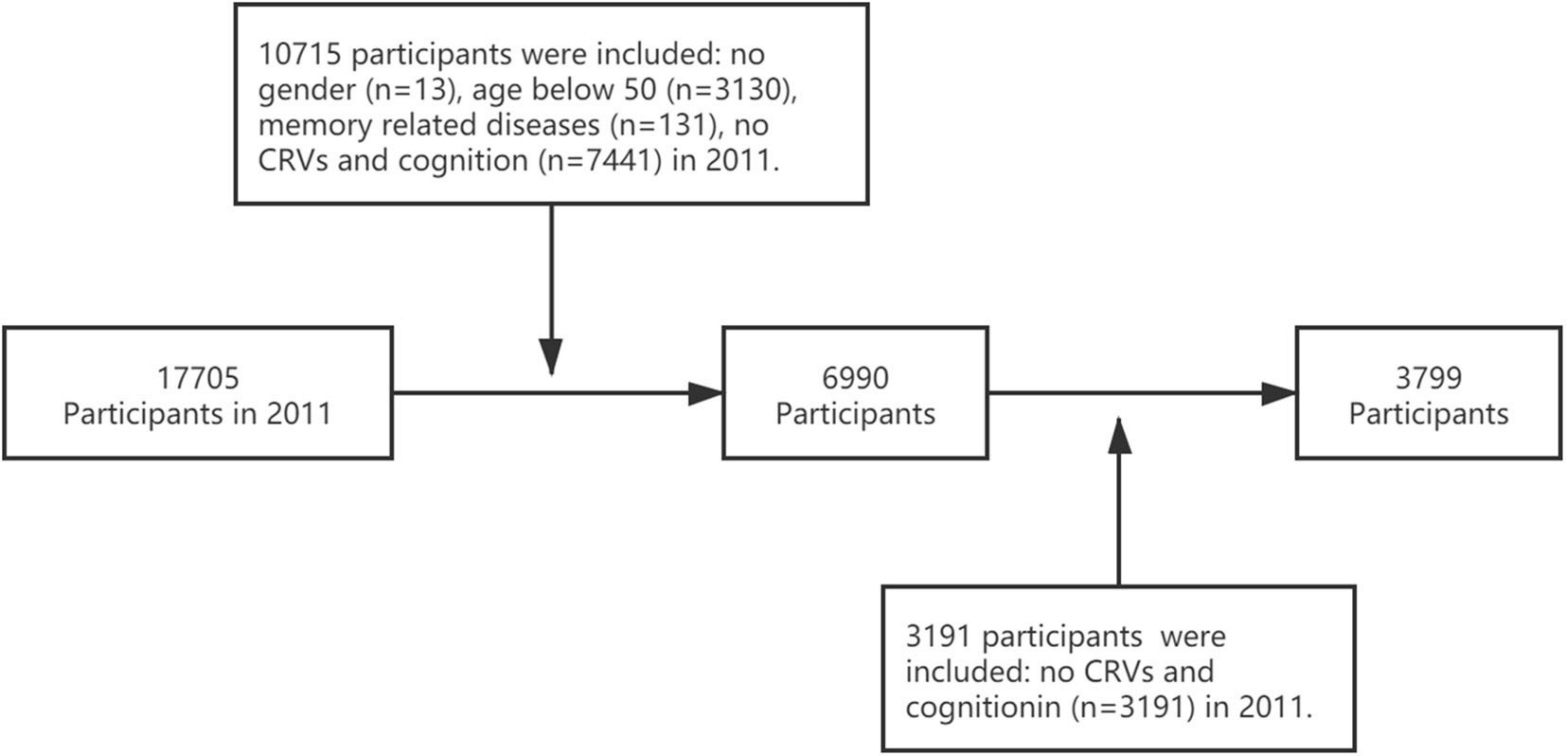
Flowchart of included participants.

Of the total sample (*n* = 17,705), 10,715 participants from the baseline were excluded because they (1) were aged <50 years old (*n* = 3,130), (2) had no fasting blood test or no results on at least one CVD factor or cognition assessment (*n* = 7,441), (3) had the memory-related disease (*n* = 131), and (4) did not report gender (*n* = 13). We excluded 3,191 participants from the 2015 visit without CVR and cognition assessment. Finally, we included a total of 3,799 participants in the final analyses (Figure 1).

About half of the target participants were female (50.6%), and the mean age was 62.12 (SD = 7.75). Besides, 84.4% of the participants possessed agricultural hukou, while only 8.3% had high school or above degrees, and 87.3% were married. Around one third lived in eastern China (developed part), and almost all participants (99.3%) resided in family houses. Also, nearly three quarters conducted farming or/and no-farming jobs.

Other detailed information for health status and behavior are also shown in Table 1.

**Table 1:** Characteristics of participants (*N* = 3,799).

	**%(n)/M ± SD**
**Demographic characteristic variables**	
Age (years)	62.12 ± 7.75
50–65	69.4 (2,638)
Above 65	30.6 (1,161)
Gender	
Male	49.4 (1,877)
Female	50.6 (1,922)
Hukou	
Agricultural hukou	84.4 (3,207)
Non-agricultural hukou	15.6 (592)
Education level	
Below high school	91.7 (3,482)
High school or above	8.3 (317)
Marital status	
Married	87.3 (3,316)
Separated, divorced, and widowed	12.0 (457)
Never married	0.7 (26)
**Socioeconomic factors**	
Regional distribution	
Eastern China	32.8 (1,245)
Mid-China	27.5 (1,045)
Western China	39.7 (1,509)
Type of address	
Family housing	99.3 (3,771)
Nursing home	0.2 (8)
Hospital	0.0 (1)
Other	0.5 (19)
Employment status	
Farming	63.4 (2,408)
Non-farming	8.4 (320)
Both farming and non—farming	0.1 (8)
None job	28.1 (1,067)
**Health and behavior variables**	
BMI	23.42 ± 3.91
WHtR	0.54 ± 08
Smoking status	
No	58.4 (2,217)
Yes	41.6 (1,582)
Alcohol status	
Drink more than once a month	26.2 (997)
Drink but less than once a month	7.4 (283)
None of these	66.3 (2,519)
HDL cholesterol (mg/dl) at 2011	51.31 ± 16.38
HDL cholesterol (mg/dl) at 2015	51.56 ± 12.57
Total cholesterol (mg/dl) at 2011	194.70 ± 39.79
Total cholesterol (mg/dl) at 2015	185.76 ± 37.02
Systolic reading at 2011	131.38 ± 25.99
Systolic reading at 2015	130.26 ± 20.62
Diabetes	
Yes	5.9 (226)
No	94.1 (3,573)
Stroke	
Yes	2.0 (77)
No	98.0 (3,722)
Depression at 2011	0.86 ± 62
Depression at 2015	0.84 ± 65
**Preliminary variables**	
CVR at 2011	8.44 ± 6.30
CVR at 2015	8.32 ± 6.47
Immediate recall at 2011	3.49 ± 2.00
Delayed recall at 2,011	2.60 ± 2.02
Orientation at 2011	3.59 ± 1.46
Calculation at 2011	2.10 ± 1.13
Figure copying at 2011	0.61 ± 49
General cognition at 2011	12.39 ± 5.32
Immediate recall at 2015	3.39 ± 1.95
Delayed recall at 2015	2.40 ± 2.02
Orientation at 2015	3.38 ± 1.63
Calculation at 2015	2.04 ± 1.16
Figure copying at 2015	0.56 ± 50
General cognition at 2015	11.77 ± 5.69

*Values are M ± SD (means ± standard deviations) for continuous variables and percentages for categorical variables. BMI, body-mass index; CVR, cardiovascular risk; HDL, high-density lipoprotein; WHtR, waist to height*.

### Association Between Composite CVR and Cognition

The model fit index (CFI) ranged from 0.984 to 0.996, RMSEA from 0.032 to 0.059, and SRMR from 0.005 to 0.013 (Table 1). More indices for model fit are also shown in Table 2.

**Table 2:** Fit indices of the various models.

	**Model description**	***χ^2^***	***df***	***χ^2^/df***	**CFI**	**TLI**	**SRMR**	**RMSEA**	**[90% CI]**	**Δ*χ2***	**ΔCFI**	**ΔTLI**
Immediate recall	Model 1a: Autoregressive model	51.43	6	8.57	0.988	0.907	0.011	0.049	[0.037, 0.062]	–	–	–
	Model 2a: CVR main-effect model	31.06	5	6.21	0.993	0.936	0.007	0.041	[0.028, 0.055]	20.37^**^	0.005	0.029
Delayed recall	Model 1b: Autoregressive model	43.79	6	7.30	0.990	0.921	0.011	0.045	[0.033, 0.058]	–	–	–
	Model 2b: CVR main-effect model	20.85	5	4.17	0.996	0.960	0.006	0.032	[0.018, 0.046]	22.94^**^	0.006	0.039
Orientation	Model 1c: Autoregressive model	45.91	6	7.65	0.990	0.923	0.009	0.046	[0.034, 0.059]	–	–	–
	Model 2c: CVR main-effect model	37.73	5	7.55	0.992	0.924	0.007	0.046	[0.033, 0.060]	8.18^**^	0.002	0.001
Calculation	Model 1d: Autoregressive model	67.61	6	11.27	0.984	0.881	0.013	0.057	[0.045, 0.070]	–	–	–
	Model 2d: CVR main-effect model	31.94	5	6.39	0.993	0.937	0.007	0.041	[0.028, 0.056]	35.67^**^	0.009	0.056
Figure copying	Model 1e: Autoregressive model	36.07	6	6.01	0.992	0.936	0.009	0.040	[0.028, 0.053]	–	–	–
	Model 2e: CVR main-effect model	20.85	5	4.17	0.996	0.959	0.005	0.032	[0.018, 0.046]	15.22^**^	0.004	0.023
General cognition	Model 1f: Autoregressive model	70.98	6	11.83	0.985	0.887	0.013	0.059	[0.018, 0.046]	–	–	–
	Model 2f: CVR main-effect model	33.55	5	6.71	0.994	0.940	0.008	0.043	[0.030, 0.057]	37.98^**^	0.009	0.053

** *p <0.01*.

**Figure 2:**
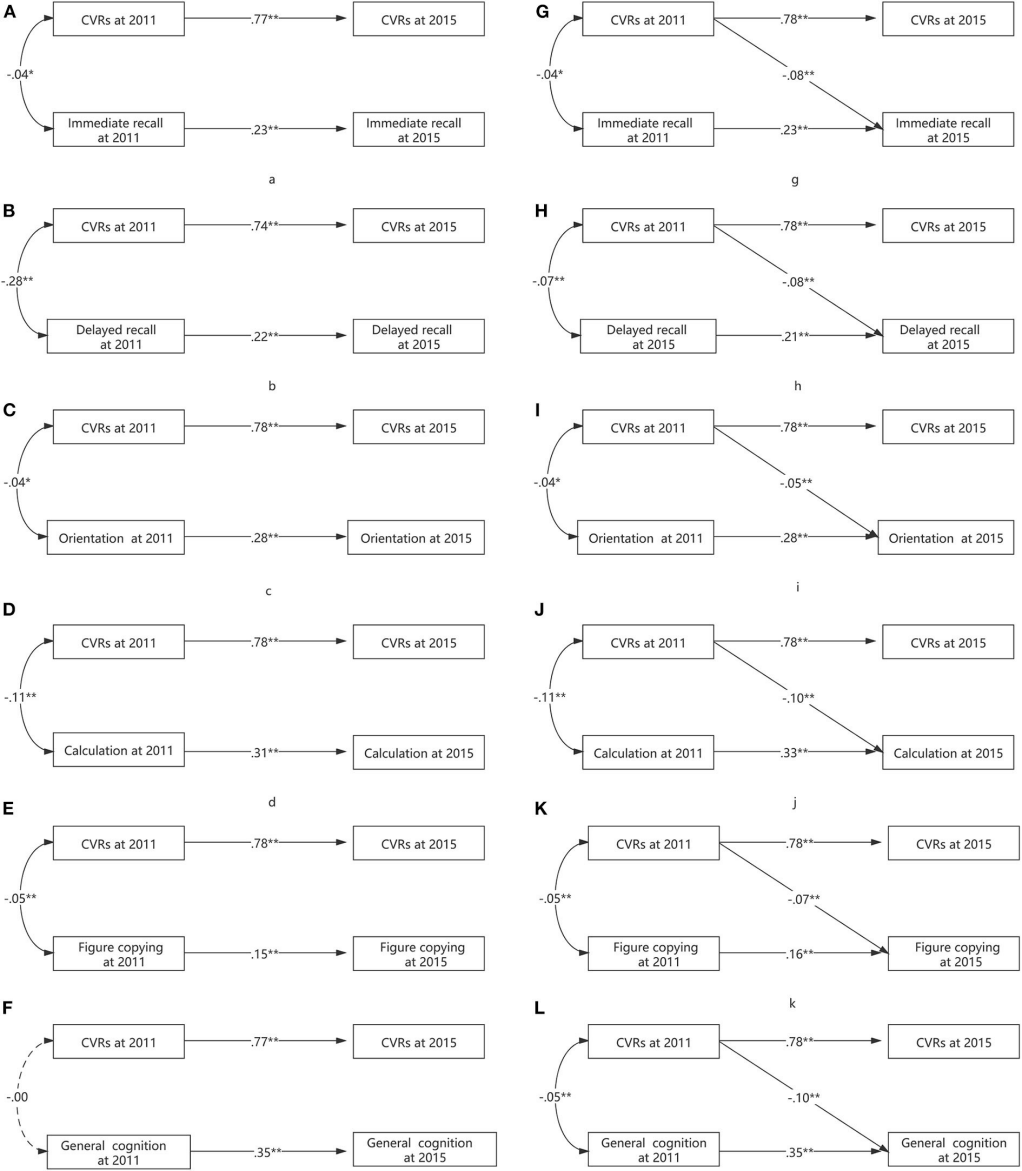
**(A–L)** The longitudinal association between CVR and cognition.

At baseline, we found that the FCRS had a consistent negative linear association with global cognition (β = −0.05, *p* < 0.01). Additionally, FCRS had inverse linear associations with domain-specific measurements of memory and learning (delayed recall and immediate recall), the calculation (100 minus 7), orientation, and visual–spatial ability (figure copying) (Figure 2).

At follow-up, we found that the baseline FCRS had an inverse linear association with follow-up global cognition (β = −0.07, *p* < 0.01). Besides, baseline FCRS had inverse linear associations with domain-specific measurements of memory and learning (delayed recall and immediate recall), the calculation (100 minus 7), orientation, and visual–spatial ability (figure copying) (Figure 2).

### Sex Modification

**Figure 3:**
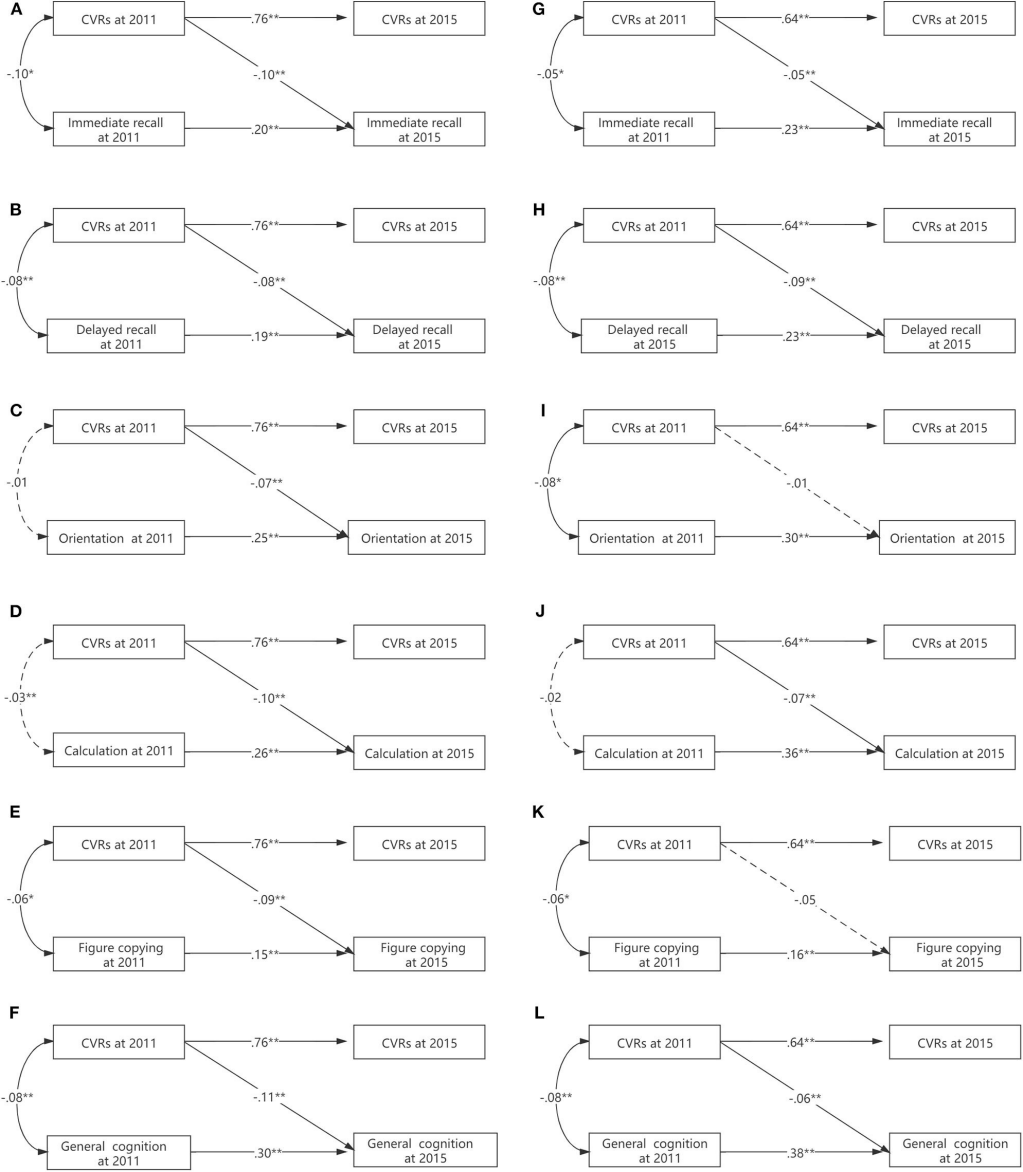
**(A–L)** The moderating effects of sex on the association between CVR and cognition.

Tests of interactions for CVR with sex provided evidence for modification, indicated by ΔCFI > 0.01 in all outcomes (Table 3). Males had a more pronounced association between higher CVR and worse general cognition, immediate recall, orientation, calculation, and visual–spatial ability (all values of *p* < 0.0001), while females exhibited a slightly larger negative association in delayed recall (Figure 3).

**Table 3:** Fit indices of the various models across sex.

	**Model description**	***χ^2^***	***df***	***χ^2^/df***	**CFI**	**TLI**	**SRMR**	**RMSEA**	***[*90% *CI]***	**Δ*χ2***	**ΔCFI**	**ΔTLI**
Immediate recall	Model 1a: Free estimation model	29.50	10	2.95	0.995	0.951	0.008	0.035	[0.021, 0.050]	–	–	–
	Model 2a: Path equivalence model	172.03	51	3.37	0.967	0.940	0.023	0.039	[0.033, 0.045]	142.53^**^	−0.028	−0.011
Delayed recall	Model 1b: Free estimation model	19.38	10	1.94	0.997	0.976	0.006	0.024	[0.006, 0.041]	–	–	–
	Model 2b: Path equivalence model	150.32	51	2.95	0.972	0.949	0.022	0.035	[0.029, 0.042]	130.94^**^	−0.025	−0.027
Orientation	Model 1c: Free estimation model	21.91	10	2.19	0.997	0.971	0.007	0.028	[0.011, 0.043]	–	–	–
	Model 2c: Path equivalence model	177.25	51	3.48	0.967	0.940	0.024	0.040	[0.033, 0.046]	155.34^**^	−0.03	−0.031
Calculation	Model 1d: Free estimation model	18.87	10	1.89	0.998	0.978	0.006	0.024	[0.004, 0.040]	–	–	–
	Model 2d: Path equivalence model	171.86	51	3.37	0.967	0.941	0.025	0.039	[0.033, 0.045]	152.99^**^	−0.031	−0.037
Figure copying	Model 1e: Free estimation model	16.23	10	1.62	0.998	0.983	0.005	0.020	[0.000, 0.037]	–	–	–
	Model 2e: Path equivalence model	142.35	51	2.79	0.973	0.951	0.023	0.034	[0.027, 0.040]	126.12^**^	−0.025	−0.032
General cognition	Model 1f: Free estimation model	32.28	10	3.23	0.995	0.952	0.008	0.038	[0.024, 0.053]	–	–	–
	Model 2f: Path equivalence model	202.43	51	3.97	0.965	0.936	0.025	0.043	[0.024, 0.053]	170.15^**^	−0.030	−0.016

** *p <0.01*.

### Age Modification

**Figure 4:**
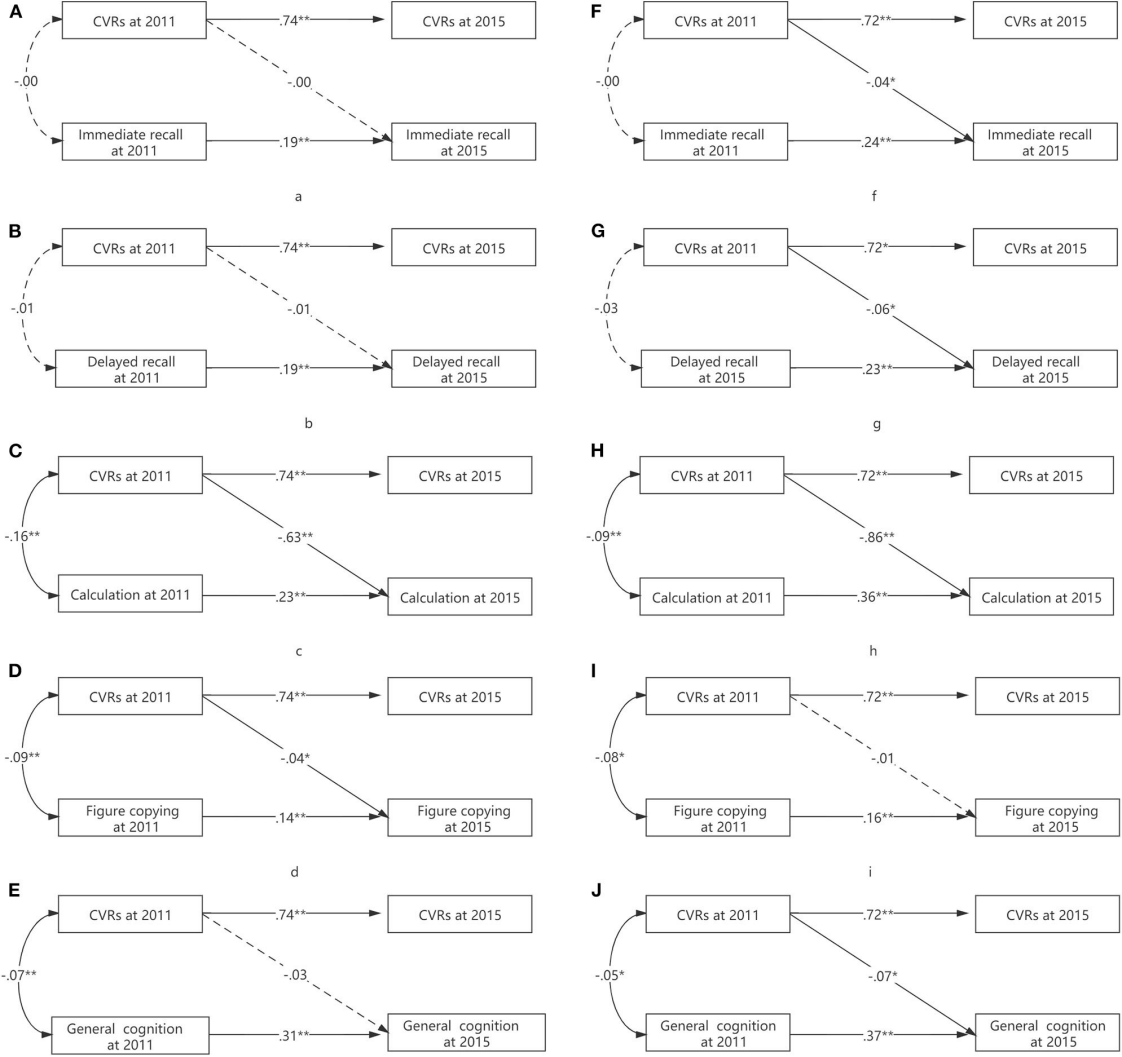
**(A–J)** The moderating effects of age on the association between CVR and cognition.

Tests of interactions for CVR with age provided some evidence for modification in the calculation test (ΔCFI = 0.11) (Table 4). Older participants (>65 years old) had a more pronounced association between higher CVR and worse calculation ability (*p* = 0.003) (Figure 4).

**Table 4:** Fit indices of the various models across age.

	**Model description**	***χ^2^***	***df***	***χ^2^/df***	**CFI**	**TLI**	**SRMR**	**RMSEA**	**[90% CI]**	**Δ*χ2***	**ΔCFI**	**ΔTLI**
Immediate recall	Model 1a: Free estimation model	38.81	10	3.88	0.991	0.916	0.008	0.043	[0.029, 0.057]	–	–	–
	Model 2a: Path equivalence model	103.91	51	2.04	0.983	0.970	0.016	0.026	[0.019, 0.033]	65.1^**^	−0.008	0.054
Delayed recall	Model 1b: Free estimation model	31.14	10	3.11	0.993	0.936	0.007	0.037	[0.023, 0.052]	–	–	–
	Model 2b: Path equivalence model	92.28	51	1.81	0.986	0.975	0.015	0.023	[0.015, 0.030]	61.14^*^	−0.007	0.039
Orientation	Model 1c: Free estimation model	64.84	10	6.48	0.984	0.853	0.010	0.059	[0.046, 0.073]	–	–	–
	Model 2c: Path equivalence model	114.78	51	2.25	0.981	0.966	0.016	0.028	[0.021, 0.035]	49.94	−0.003	0.113
Calculation	Model 1d: Free estimation model	47.86	10	4.79	0.989	0.898	0.009	0.049	[0.036, 0.063]	–	–	–
	Model 2d: Path equivalence model	126.96	51	2.49	0.978	0.960	0.018	0.031	[0.024, 0.038]	79.1^**^	−0.011	0.062
Figure copying	Model 1e: Free estimation model	32.26	10	3.23	0.993	0.932	0.007	0.038	[0.024, 0.053]	–	–	–
	Model 2e: Path equivalence model	90.58	51	1.78	0.987	0.976	0.015	0.022	[0.015, 0.030]	58.32^*^	−0.006	0.044
General cognition	Model 1f: Free estimation model	53.53	10	5.35	0.989	0.895	0.010	0.053	[0.039, 0.067]	–	–	–
	Model 2f: Path equivalence model	127.61	51	2.50	0.980	0.964	0.017	0.031	[0.024, 0.053]	74.08^*^	−0.009	0.069

* *p <0.05*;

** *p <0.01*.

## Discussion

We found that higher CVR was associated with cognitive decline in various cognitive tasks. These results extended the evidence from other ethnicities (8, 9, 22) to Chinese. Additionally, existing evidence drives mainly from young older adults. Our sample fixed this limitation by recruiting the participants with a wide range of age spectrum (50–93 years old). We also found age and sex modification effects on multiple cognitive tasks but in different patterns: age modification effects on the calculation test while sex modification effects on all tests.

Regarding the association between CVR and cognitive function, our results were consistent with previous original articles in older adults (27–31). The association remains significant even after adjusting other demographic and socioeconomic covariates, indicating CVR might be an independent factor for cognition. Besides, our results found the negative association between CVR and all cognitive tasks. A recent fMRI study comprising 9,722 participants found that CVR was associated with higher white matter hyperintensity volumes, poorer white matter microstructure in association and thalamic pathways, and lower frontal, temporal volumes (32). This large-scale brain atrophy may be the cause of the cognitive decline in multiple cognitive domains. Also, CVD (e.g., hypertension) may lead to cerebral hypoperfusion, which may also trigger cascade impacts on various brain regions (33).

Regarding the modification effects of sex, sex modified all cognitive tasks. Sex differences were observed in global cognitive function (34, 35) and in several cognitive domains (e.g., episodic memory and executive function) among older adults (36–38). Besides, sex differences were also overserved in CVD's prevalence or incidence (39–41), indicated by higher CVD in men. Middle-aged and older Chinese have a much higher probability of abdominal obesity in men and more likely to meet the criteria of metabolic syndrome (42). Interestingly, Asperholm et al. (37) conducted a meta-analytic study examining sex differences in various episodic memory tasks and found that men may use their spatial advantage in spatially episodic memory tasks. In contrast, women do well in episodic memory tasks that are verbalized recall and non-verbal and non-spatial tasks that are neither verbal nor spatial. Though there are sex differences in both CVD and cognitive function, the role of sex in the relationship between CVD and cognition is complex and requires additional investigation.

With respect to the modification effects of age, the aging process is associated with altered brain intrinsic connectivity (43) and brain atrophy (44). The aging process is also associated with cognitive decline in multiple cognitive domains (45, 46). The age and CVR interaction in predicting cognitive function also partially replicated in other ethnicity (22). Old-old participants demonstrated a steeper slope in the calculation test than the young-old, which means calculation ability might be a sensitive behavior marker in evaluating the age moderation. Therefore, calculation training programs should be adopted as preventive strategies in middle-aged and young-old adults and mitigating methods for the old-old.

The study has several strengths. First, it used CHARLS data, the most representative and technically sound cohort with the largest sample size. Second, we adopted a composite CVR score to elucidate the association, which provided a relatively comprehensive picture of this issue. Third, the longitudinal study offers us opportunities to evaluate the temporal relationship between CVR and cognition.

Several limitations should also be addressed. First, we could not link genetic risk factors (e.g., APOE) with cognitive function. APOE plays a vital role in lipid metabolism and amyloid pathology cognitive function (47, 48). Second, we did not collect MRI data. A recent study using UK Biobank data has identified that the CVD is associated with brain atrophy in large-scale brain regions (32). These limitations should be solved in future megacohorts by comprehensively evaluating the risk profiles. Third, we did not adopt an in-depth multimodality cognitive assessment. Other cognitive domains (e.g., complex attention or executive function) should also be taken into consideration. Fourth, the rate of loss-to-follow is high, which may lead to selection bias. Participants in worse conditions at baseline may be more likely to drop out of the cohort due to illness or mortality in the follow-up visits.

## Conclusion

CVD are risk factors for lower global cognitive function and subdomains in middle-aged and older adults in China. Sex significantly moderates the CRV–cognition associations while age only moderating the CRV–calculation association.

## Data Availability Statement

The datasets presented in this study can be found in online repositories. The names of the repository/repositories and accession number(s) can be found at: http://charls.pku.edu.cn/index/en.html.

## Ethics Statement

The studies involving human participants were reviewed and approved by Institutional Review Board of Beijing Youan Hospital. The patients/participants provided their written informed consent to participate in this study.

## Author Contributions

JH, WH, and WW were responsible for the study concept and design. JH, RS, and BC analyzed the data. JH, JF, RS, CG, and WW interpreted the data. JH drafted the manuscript. All authors revised and approved the manuscript.

## Conflict of Interest

The authors declare that the research was conducted in the absence of any commercial or financial relationships that could be construed as a potential conflict of interest.
