# Supplementary material for: The Longitudinal Association Between Cardiovascular Risk and Cognitive Function in Middle-Aged and Older Adults in China: A Nationally Representative Cohort Study
Source: Front Cardiovasc Med. 2020 Oct 19;7:560947. doi: 10.3389/fcvm.2020.560947 (PMC7604338; doi:10.3389/fcvm.2020.560947)
Supplement: Supplementary file 1 [file Data_Sheet_1.doc]

**Informed consent of China Health and Retirement Longitudinal Survey(CHARLS)**

You are invited to participate in this survey because you are an eligible respondent of CHARLS. Please read the informed consent carefully and decide whether or not to participate in the survey. When the interviewer discusses the informed consent with you, you can ask him or her to explain what you do not understand. We encourage you to discuss with your family and friends adequately before you agree to participate in the survey. The content/nature, risks, inconvenience, discomfort and other important information of the survey are as follows:

This survey is supervised by Professors Yaohui Zhao of the National School of Development of Peking University and Yang Gonghuan of the China Center for Disease Control and Prevention (China CDC).

1. **Why do we conduct this survey?**

The rapid population aging is an increasingly prominent problem in China. The academic research on aging issues is still in primitive in China and there is an urgent need to collect a high quality and multidisciplinary data set to provide the basis of aging research. The survey aims at collecting information on health and retirement of China residents aged 45 and older, which can assist the making of national social security policies on health andretirement, and promote interdisciplinary research on aging issues of China.

1. **Who will participate in the survey?**

Our study is being conducted in 150 counties or cities districts in 28 provinces. Three administrative villages or urban communities are selected randomly in each county with probability proportionate to size sampling (PPS) method. iIn every chosen village or community we aim to select 24 eligible households randomly. In every chosen household, one member aged 45 or older is selected randomly. He/she and the spouse may voluntarily participate in our study.

1. **What is the content of the survey?**

If you choose to participate in the study, our interviewer will first ask you about your basic demographics, family information, health status, health care and insurance, work, retirement and pensions, household and individual income, expenditure, and assets, etc. Then the interviewer will give you a physical examination to better understand your true health conditions. The measurements include height, weight, waist circumference, blood pressure, peak meter flow lung capacity, grip strength, balance, timed walk, and timed sit to stand, and draw 8-ml (about one and half spoons) of blood for routine blood tests and test for Total Cholesterol, High Density Lipoprotein, HbA1c and C-reaction Protein. Roughly after three weeks of the blood tests we will inform you to obtain the blood routine examination results and the need for follow-up health counseling.

1. **How long will the survey take?**

It takes about 120-150 minutes to complete the survey. And we will do the follow-up survey every two years. You may withdraw from the study any time, which will not impact any of your benefits.

1. **What is the risk of participating in the survey?**

Generally, the study will not cause injury. The blood drawing procedure will not transmit diseases to you, because the syringe and needle are new and disinfected. The small amount of blood drawn has no harm to your health. Withdrawing blood may cause very mild, local pain, and/or bruise or swelling, and, in extremely rare cases, fainting. While waiting for your test results, you may feel anxious. If you find out that your test results are abnormal, you may be upset and fearful. Our staff will help you deal with these feelings and the questions you may have. After completing the tests required by this study, your remaining blood samples will be kept in the China CDC. It may be used for further health related tests. Your blood samples will be used only for research; it will not be sold or used to manufacture commercial products. We will notify you if the test results have direct implications for your health.

1. **What are the benefits of participating in the survey?**

If you agree to participate in the survey, the physical examination and test results related to your health will feed back to you directly. And the information you provided can be used to help make health, retirement and social security policies suitable for China, which will benefit you and other older people just like you.

1. **Will my information be keep confidential?**

We will abide by the law and keep your survey information confidential. We will use computer aided interview system to record your questionnaires and physical examination information. All your personal records including fingerprint, questionnaires, and physical examination and test results are confidential; we will not tell others, include your family, friends, local hospitals, etc. Unless required by law, your personal information, including name , identity card number, address, phone number, and other information which can be used to identify you will not be disclosed. you are identified by a number in the questionnaires and test records, which will be stored safely in CHARLS project office.

The staff members working on this study and the persons in charge of reviewing the study (sponsors and the Biomedical Ethics Review Committee in Peking University) will be able to see only the coded number on your records, but not your name. Only a few designated investigators will know your name. All published research will not include your personal identification.

1. **Is there a few for participating in the survey?**

If you agree to participate in this study, all the interviews, physical examination, tests and counseling are provided to you for free. You do not need to pay anything.

1. **What is the compensation for me?**

If you agree to participate in this study, you will get a compensation for your time and possible travel. The compensation standard is as follows: conditional on finishing all survey work (questionnaire interview, physical examination, blood test and sample storage), if the household has one respondent then payment is 30 Yuan and higher; if the household has two respondents (the age eligible respondent and the spouse) the payment will bet at least 50 Yuan.

1. **What is the compensation for injuries related to the survey?**

In case you are injured as a direct result of your participation in this study, China CDC and Peking University will give you immediate and necessary treatment for your injuries which will be free to you, and you will not be compensate financially .

1. **The right to refuse and withdraw from survey**

You may withdraw from the study any time, which will not impact any of your benefits. In principal, after you withdraw from the survey, the researcher will keep your information confidentially until it is destroyed, and your information will not be used or disclosed during this period.

1. **Who should I contact if I have questions or problems?**

If you have any questions about this study, you may contact Prof. Yaohui Zhao at Peking University at 400-610-1866 or 010-62754803.

If you have any questions about your right or benefit, or you want to report a problem, complaint and worries encountered during the survey, or you want to offer advice and suggestions related to the survey, please contact Biomedical Ethics Review Committee of Peking university at 010-82801426 or by email [llwyh@bjmu.edu.cn](mailto:llwyh@bjmu.edu.cn).

**Statement by the interviewer taking the informed consent**

“I have informed the respondent about the background, goals, procedure, risks and benefits of the survey, given him/her enough time to read the informed consent and discuss with others, and answered all questions related to the survey; I have informed the respondent that he/she can contact the project Director, Professor Yaohui Zhao, when having problems about the survey and contact Biomedical Ethics Review Committee of Peking university when having problems about his/her right or benefits anytime, and provided the accurate contact information. I have informed the respondent that he/she can withdraw from the survey anytime. I have informed the potential respondent that he/she can get a copy of this informed consent with signatures of mine and his/hers.

_____year ___month __date

Interviewer’s signature Date

**Statement by the respondent**

“I was informed about the background, goals, procedure, risks and benefits of the survey. I have enough time and opportunity to ask questions and I was satisfied with the answers. I was informed who I can contact when I have questions, difficulty, worries, or suggestions, or want to know more about or help the survey. I have read the informed consent and agree to participate in the survey. I know I can withdraw from the survey anytime even without any reason. I have been informed that I can get a copy of this informed consent with the signatures of mine and the interviewer’s.

**I agree to participate in**

**□questionnaire survey**

**□physical examination**

**□blood test**

**□blood storage**

**Sign here:**

________ ________ __year__month__date

Respondent’s name(print or written clearly) respondent’s signature or finger print Date

________ ________ __year__month__date

Proxy’s name (print or written clearly) Proxy’s signature Date

The relation with the respondent_________________
